# “I've Always Thought That I Was Not Good at Experiments…”—The Benefit of Non-formal Learning in Terms of Students' Perceived Competence

**DOI:** 10.3389/fpsyg.2022.882185

**Published:** 2022-05-17

**Authors:** Tim Kirchhoff, Matthias Wilde, Nadine Großmann

**Affiliations:** Faculty of Biology, Biology Didactics, Bielefeld University, Bielefeld, Germany

**Keywords:** non-formal learning, science outreach program, perceived competence, experimentation, biology education

## Abstract

Outreach science labs have been established as non-formal out-of-school learning environments in the fields of science, technology, engineering, and mathematics. Previous research has suggested that visiting an outreach science lab can be beneficial in terms of student motivation. Nevertheless, the current research on these out-of-school learning environments lacks studies that investigate important variables for the development of self-determined student motivation, such as perceived competence. In our study, we investigated the moderating effect of the learning environment on the relationship between students' contextual competence perceptions and their situational competence experiences regarding experimentation. For this purpose, 119 students in the first year of the upper secondary school participated in an experimental course on enzymology at an outreach science lab (*n* = 60) and in their biology classroom at school (*n* = 59). Our results showed that the relationship between students' contextual competence perceptions and their situational competence experiences during experimentation is moderated by the learning environment. The analyses revealed that students with a higher contextual competence perception showed comparable situational experiences of competence in both learning environments. In contrast, the students who perceived themselves as less competent at a contextual level benefited from experimenting at the outreach science lab in terms of their situational competence experiences.

## Introduction

Motivation and interest play a key role in student learning and choice of profession (Bennett and Hogarth, 2009; Gottfried et al., 2009; Archer et al., 2010; Krapp and Prenzel, 2011; Organisation for Economic Co-operation and Development (OECD), 2016, 2019; Ryan and Deci, 2017). However, Programme for International Student Assessment studies have demonstrated that students lose their science-related motivation and interest through the course of their education (Krapp and Prenzel, 2011; Organisation for Economic Co-operation and Development (OECD), 2016, 2019). With regard to an increasing demand for graduated scientists and employees in the fields of science, technology, engineering, and mathematics (STEM; Organisation for Economic Co-operation and Development (OECD), 2016; Bureau of Labor Statistics and Department of Labor, 2017; Statistik der Bundesagentur für Arbeit, 2019), this negative trend in student motivation is a cause of concern and needs to be addressed in science education.

As a result, many studies have focused on out-of-school learning in science educational outreach programs in recent years (e.g., Salmi, 2012; Schütte and Köller, 2015; Clark et al., 2016; Roberts et al., 2018; Tsybulsky et al., 2018; Tal and Dallashe, 2019; Tsybulsky, 2019; Maiorca et al., 2021; Solis et al., 2021). To complement formal science learning at school, informal and non-formal science learning can make valuable contributions to student learning due to, among other things, authentic hands-on learning experiences (Tal and Dallashe, 2019; Maiorca et al., 2021). Science learning in informal and non-formal contexts can be beneficial for student engagement in science classes (Roberts et al., 2018), the acquisition of skills and knowledge (Berg et al., 2021; Solis et al., 2021), and the promotion of student motivation and interest in the fields of STEM (Mohr-Schroeder et al., 2014; Schütte and Köller, 2015; Clark et al., 2016; Tal and Dallashe, 2019). Against this backdrop, outreach science labs (OSLs) have been established in Germany. In these out-of-school learning environments, students can perform experiments in an authentic science laboratory (Scharfenberg et al., 2019; Euler and Schüttler, 2020). It has been assumed that performing experiments at an OSL can be beneficial to students' science-related motivation and interest (Hofstein and Lunetta, 2004; Scharfenberg et al., 2019; Euler and Schüttler, 2020).

Previous studies have generally painted single visits to an OSL in a positive light (for an overview see Guderian and Priemer, 2008; Nickolaus et al., 2018; Scharfenberg et al., 2019). For instance, the characteristics of an OSL, including its authenticity, the laboratory work, and the supervision, have been found to have a positive impact on students' situational interest (Pawek, 2009) and situational competence experience (see also Glowinski, 2007; Glowinski and Bayrhuber, 2011; Itzek-Greulich and Vollmer, 2017). Students' situational competence experience was also found to be affected by their self-concept of abilities (Glowinski and Bayrhuber, 2011; Damerau, 2012) and contextual competence perception (i.e., a more habitual competence perception; Kowal and Fortier, 2000; Milyavskaya et al., 2013). However, the effects of these more habitual motivational variables on situational motivational outcomes may depend on the learning environment (see Glowinski and Bayrhuber, 2011; Itzek-Greulich and Vollmer, 2017; Scharfenberg et al., 2019).

Situational competence experience is an important antecedent of self-determined motivation (Reeve, 2015; Ryan and Deci, 2017; Ryan and Moller, 2017). Therefore, investigating predictors of students' situational competence experience at OSLs and at school is of unique importance for the understanding of both motivational processes during laboratory work and the effectiveness of OSLs in terms of student motivation (see Itzek-Greulich and Vollmer, 2017; Nickolaus et al., 2018). However, the current research in this field lacks studies that consider a school treatment under more realistic conditions (e.g., using equipment that is usually available at schools) and the same pedagogical staff to conduct the same educational program in both settings (see Itzek-Greulich and Vollmer, 2017; Nickolaus et al., 2018; Tsybulsky, 2019; Röllke et al., 2020). The current study addresses this research gap. The same educational program was conducted by the same pedagogical staff at an OSL and at school using equipment that is usually available in the respective learning environments. In these learning environments, we investigated the moderating effect of the learning environment on the relationship between students' contextual competence perception and situational competence experience during experimentation.

## Theory

### The Formality of Out-of-School and In-school Learning

Student science learning is not limited to traditional learning at school, but may also take place in out-of-school contexts (Eshach, 2007); for instance, it may occur during leisure activities or during field trips to out-of-school learning sites, such as museums, zoos, science centers, and OSLs. The various contexts of science learning are described using the terms *formal learning, informal learning*, and *non-formal learning* (Colley et al., 2003, 2006; Eshach, 2007; Stecher et al., 2018). *Formal learning* takes place intentionally in educational institutions (e.g., schools and universities) to qualify the students for a professional career (Colley et al., 2003, 2006; Eshach, 2007; Stecher et al., 2018). It is graded, certified, curriculum-based, and structured in terms of explicit learning goals and time (Malcolm et al., 2003; Colley et al., 2006; Eshach, 2007; Stecher et al., 2018). Moreover, pedagogical support is provided by a teacher (Malcolm et al., 2003; Colley et al., 2006; Eshach, 2007; Stecher et al., 2018). *Informal learning* is spontaneous, non-educational learning that can occur anywhere (Colley et al., 2003, 2006; Eshach, 2007). Compared to formal learning, activities in which informal learning occurs are not primarily intended for learning (Colley et al., 2003, 2006); for instance, such activities can be work-, hobby-, or leisure-related (Colley et al., 2003, 2006). Informal learning is neither evaluated nor certified; furthermore, it does not follow a curriculum and is not structured in terms of explicit learning goals and time (Colley et al., 2003, 2006; Malcolm et al., 2003; Stecher et al., 2018). Pedagogical support during informal learning can be provided by peers or colleagues (Colley et al., 2003, 2006). *Non-formal learning* is used as “an intermediate category” (Colley et al., 2006, p. 57) between formal and informal learning (Eshach, 2007; Werquin, 2010). It refers to intended out-of-school learning and is less formal than traditional learning at school. That is, non-formal learning is usually not graded and somewhat structured, for example, on the basis of learning goals that can be curriculum-related (Colley et al., 2006; Eshach, 2007; Stecher et al., 2018). During non-formal learning at an out-of-school learning environment, students can receive pedagogical support from local staff (e.g., guides, mentors, and tutors; Colley et al., 2006). Out-of-school learning, for instance, during a field trip, can be characterized as informal or non-formal (see Colley et al., 2003; Eshach, 2007). In the following section, we discuss out-of-school learning at OSLs in the context of non-formal learning.

### Non-formal Learning at Outreach Science Labs

In Germany, an OSL is a science laboratory at a university or research center that provides science educational outreach programs for school classes (Scharfenberg et al., 2019; Euler and Schüttler, 2020). In these out-of-school learning environments, students perform hands-on laboratory activities with laboratory equipment that is usually unavailable at schools (Scharfenberg and Bogner, 2013; Garner et al., 2014; Schüttler et al., 2021). These laboratory workshops mostly follow a clear structure, relate to natural science curricula in physics, chemistry, and biology, and follow explicit learning goals. OSLs aim to provide insights into activities and career profiles in the fields of STEM and to promote students' science-related interest and motivation (Garner et al., 2014; Affeldt et al., 2015; Scharfenberg et al., 2019; Euler and Schüttler, 2020). Comparing subject-specific laboratories in fields of natural science, these laboratories emphasize different focal points and differ with regard to their educational programs, concepts, and goals. For instance, in the fields of physics and chemistry, OSLs primarily emphasize the creative and innovative aspects of research and development activities, as well as their importance to society (Euler and Schüttler, 2020). In the fields of biology, OSLs mainly focus on teaching methodological skills, such as practical laboratory work (Euler and Schüttler, 2020). Regarding pedagogical support, at an international level, there are science educational outreach programs in which the local staff (e.g., “Bristol ChemLabS,” Shallcross et al., 2013; “PHIRE,” Hanauer et al., 2006; “Medical Simulation-Based Environment,” Tal and Dallashe, 2019) or the students' regular teachers lead the laboratory workshop and provide pedagogical support (e.g., “UHasselt@school,” Guedens and Reynders, 2012; “teacher-led outreach laboratories,” Stolarsky Ben-Yun and Yarden, 2009). However, in German OSLs, students are usually supervised by research assistants or university student assistants (tutor; Garner et al., 2014; Scharfenberg et al., 2019; Euler and Schüttler, 2020). The tutors provide additional instructions, explanations, and guidance (Scharfenberg and Bogner, 2013, 2016). The tutor–student ratio at an OSL is more balanced than the teacher–student ratio at school. One tutor supervises a small group of students and is not responsible for an entire class in the same way that a teacher at school would be (Pawek, 2009; Scharfenberg and Bogner, 2013; Garner et al., 2014). In addition, students' performance at an OSL is generally not graded (Pawek, 2009; Glowinski and Bayrhuber, 2011; Itzek-Greulich and Vollmer, 2017).

The non-formal atmosphere of an OSL may provide multifaceted and intensive experiences (see Hofstein and Lunetta, 2004; Mohr-Schroeder et al., 2014; Roberts et al., 2018), including enjoyable, entertaining learning experiences (e.g., Tal and Dallashe, 2019; Tsybulsky, 2019) that can be described as “valuable for its own sake, regardless of the presence or absence of learning outcome” (Packer, 2006, p. 341; see also Schwan et al., 2014). These motivational experiences are depicted in various related motivational constructs, such as intrinsic motivation (Ryan and Deci, 2017), flow (Csikszentmihalyi, 2000; Engeser et al., 2021), and situational interest (Renninger and Hidi, 2016). Indeed, several studies have found positive effects of an OSL visit on students' situational interest (e.g., Itzek-Greulich and Vollmer, 2017; Schüttler et al., 2021; for an overview, see Guderian and Priemer, 2008; Nickolaus et al., 2018; Scharfenberg et al., 2019). Itzek-Greulich and Vollmer (2017) suggested that the added value of OSLs might be that these laboratories provide opportunities to conduct hands-on experiments, which are seldom implemented or not implemented at all in schools. They also found that the differences in the learning environment (laboratory vs. school) were less important to students' interest, but more important in their situational competence experience. Previous studies have also found that visiting an OSL positively impacts students' situational competence experience (e.g., Glowinski and Bayrhuber, 2011). Perceived competence plays a key role in motivational processes (Krapp, 2005; Ryan and Deci, 2017) and is explored in detail in the following section.

### Perceived Competence

*Perceived competence* is an integral element of many motivational concepts, such as competence motivation (White, 1959), self-efficacy (Bandura, 1977), self-concept (Shavelson et al., 1976), and the basic psychological need for competence (Ryan and Deci, 2017; for an overview, see Elliot et al., 2002; Hughes et al., 2011; Marsh et al., 2017). A common element of these concepts and theories is the assumption that individuals want “to acquire competence and avoid incompetence” (Elliot et al., 2002, p. 361). In line with White (1959), self-determination theory fruitfully combines the perception of competence and motivation (Elliot et al., 2002; Ryan and Moller, 2017). In this theory, competence is anchored as an innate psychological need (Ryan and Deci, 2017). This need refers to individuals' endeavor to experience themselves as effective and able to overcome challenges (Reeve, 2015; Ryan and Deci, 2017; Ryan and Moller, 2017). The perception of having control over one's actions as well as a match between one's perceived skills and challenges associated with the action are preconditions for experiencing competence (Reeve, 2015; Ryan and Deci, 2017). Thus, engaging in tasks that correspond to one's perceived skills can have a positive effect on an individual's perceived competence (Jang et al., 2010; Reeve, 2015). If the challenge is too difficult or individuals feel highly controlled, individuals may presumably perceive themselves as less competent. In educational contexts, teachers can provide support (i.e., scaffolding; Vygotsky, 1978), such as prompts, instructions, and explanations, to adapt a particular challenge to an individual's skills and compensate for missing skills (Jang et al., 2010; Arnold et al., 2017; Bruckermann et al., 2017). The support aims to allow students to perceive a sense of control over the action, act autonomously, and perceive themselves as competent (Jang et al., 2010; Reeve, 2015; Ryan and Moller, 2017; Großmann et al., 2020).

Competence can be perceived on different levels (Kowal and Fortier, 2000; Vallerand and Ratelle, 2002; Milyavskaya et al., 2013; Ryan and Moller, 2017). On a *general level*, perceived competence refers to a general, rather unspecific perception of competence in life (Milyavskaya et al., 2013; Ryan and Moller, 2017). It consists of perceptions of competence in diverse contexts, episodes, and situations in an individual's life (Milyavskaya et al., 2013). On a *contextual level*, perceived competence refers to perceptions of competence in more specific contexts or domains (Kowal and Fortier, 2000; Milyavskaya et al., 2013), such as specific subjects (e.g., biology) or actions in a science class (e.g., experimentation; see Ryan and Moller, 2017). *Contextual competence perception* depends on the extent to which individuals perceive themselves as competent in a particular context or domain (Milyavskaya et al., 2013). On an *episodic level*, perceived competence describes perceptions of competence in a specific episode of life or temporal period (Milyavskaya et al., 2013). On a *situational level*, perceived competence refers to competence experience during a certain activity in a present or previous situation (Kowal and Fortier, 2000; Milyavskaya et al., 2013; Ryan and Moller, 2017). In science class, students' *situational competence experience* can refer to a situation, such as performing experiments in a short sequence of lessons (see Ryan and Moller, 2017). The abovementioned levels of perceived competence combine to make up a hierarchical model (e.g., Kowal and Fortier, 2000; Vallerand and Ratelle, 2002; Milyavskaya et al., 2013). Perceived competence at lower levels can affect perceived competence at higher levels (i.e., a bottom-up effect; Milyavskaya et al., 2013). For instance, students' competence experience in recent science lessons could serve to build up their contextual perception of competence in science class, which could further enhance their general perception of competence in school and life (Milyavskaya et al., 2013). However, bottom-up effects of situational competence experiences are rather expected in long-term. Single or short-term experiences may not have a significant impact on more habitual perceptions of competence, such as on the contextual level (see also Conway and Pleydell-Pearce, 2000; Milyavskaya et al., 2013). Inversely, perceived competence at higher levels can affect perceived competence at lower levels (i.e., a top-down effect; Kowal and Fortier, 2000; Milyavskaya et al., 2013; Großmann et al., 2020). Students who generally perceive themselves as less competent may likely experience themselves as less competent in specific contexts or situations. For instance, students' perceptions of competence on a contextual level, such as in experimentation, can influence their situational experiences during specific situations in science class (see Vallerand and Ratelle, 2002; Milyavskaya et al., 2013).

Previous studies have shown that perceived competence at different levels can serve as a predictor for student motivation, such as intrinsic motivation (see Ryan and Deci, 2017), flow (Kowal and Fortier, 1999, 2000), and situational interest (Krapp, 2005; Scharfenberg et al., 2019). Students who feel competent are more motivated to learn voluntarily and out of interest and enjoyment (Elliot et al., 2002; Ryan and Deci, 2017; Ryan and Moller, 2017), an important prerequisite for successful academic performance and achievement (Guay et al., 2003; Ryan and Deci, 2017). As described above, students' perceived competence can be affected by the provision of support, which differs between OSLs and school (section Non-formal Learning at Outreach Science Labs). This particular relationship is elaborated upon in the following section.

### The Effects of an Outreach Science Lab on Students' Perceived Competence

As explained in the previous section, the provision of support can positively influence students' competence experience. However, the provision of support differs between OSLs and schools in terms of supervisor–student ratio (Pawek, 2009; Scharfenberg and Bogner, 2013; Garner et al., 2014). Thus, a tutor at an OSL is able to pay more attention to individual students and provide support more frequently (see Pawek, 2009; Garner et al., 2014). At school, students may receive less attention and support from their teacher (Pawek, 2009; Garner et al., 2014) and may have to wait for the teacher's attention, for instance, when several (groups of) students need help at the same time. Support that aims to reduce the perceived challenges and compensate for missing skills can be particularly helpful for students who have fewer skills or who feel less competent in experimentation on a contextual level (see Vygotsky, 1978; Schmidt-Weigand et al., 2008; Itzek-Greulich and Vollmer, 2017, 2018; Großmann and Wilde, 2019). Presumably, those students and their situational competence experience may particularly benefit from the more individual and frequent support at an OSL (see Pawek, 2009; Jang et al., 2010; Reeve, 2015).

Moreover, students' experiences may be influenced by the less formal atmosphere of the field trip to an OSL (see Hofstein and Lunetta, 2004; Itzek-Greulich and Vollmer, 2017). At an OSL, students can perform experiments and give themselves a try, regardless of grading or their previous experiences in science class (see Pawek, 2009; Euler and Schüttler, 2020). Such environments may positively affect students' motivational experiences and perceived competence (see Meece et al., 2006; Itzek-Greulich and Vollmer, 2017; Alp et al., 2018). Studies have shown that this may be particularly the case for less interested students (e.g., Pawek, 2009; Damerau, 2012) or low achieving students (Itzek-Greulich and Vollmer, 2017). Presumably, those students rate their skills low and feel less competent at a contextual level or within a specific domain (see Marsh et al., 2005; Hughes et al., 2011; Möller and Trautwein, 2015). Therefore, at an OSL, students who generally feel less competent may perceive themselves as more confident when experimenting and performing the experiments with appropriate support. Altogether, these assumptions lead to our hypotheses.

## Hypotheses

**Figure 1:**
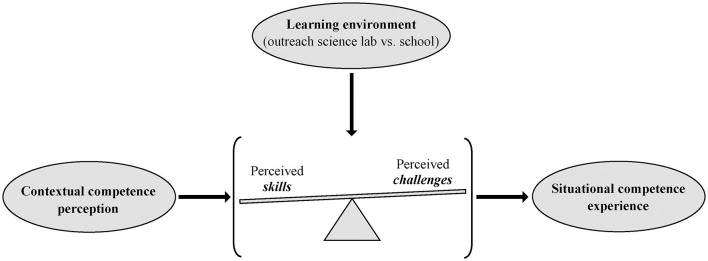
The moderating effect of the learning environment on the interrelationship of contextual competence perception and situational competence experience.

Based on the aforementioned rationale, students' situational competence experience during an experimental workshop may be affected by more habitual perceptions of competence, such as their contextual competence perception (section Perceived Competence). However, the place of learning may have an effect as well. The support provided and further characteristics of OSLs may facilitate positive experiences of competence when performing experiments (section Non-formal Learning at Outreach Science Labs and The Effects of an Outreach Science lab on Students' Perceived Competence). Previous research suggests that a visit to an OSL may be more effective for less motivated and low achieving students (e.g., Damerau, 2012; Itzek-Greulich and Vollmer, 2017). Since these variables are related to perceived competence on a contextual level (Hughes et al., 2011; Marsh et al., 2017), students who generally feel less competent in experimentation may also benefit from visiting an OSL. Specifically, we assume that the students' situational competence experience might be less dependent on their contextual competence perception at an OSL than at school. That is, the influence of students' contextual competence perception on their situational competence perception might be moderated by the learning environment (Figure 1).

In this study, we assessed students' perceived competence during two phases of their laboratory work—namely, conducting and evaluating experiments (see Klahr, 2000; Schreiber et al., 2009). Therefore, the moderating effect of the learning environment was investigated in two hypotheses, each based on one of the two phases.

H_1_: The effect of students' contextual competence perception in conducting experiments on their situational competence experience while conducting experiments is moderated by the learning environment.
H_2_: The effect of students' contextual competence perception in evaluating experiments on their situational competence experience while evaluating experiments is moderated by the learning environment.

## Materials and Methods

### Sample

We taught 119 students (71% female) in the first year of the upper secondary school (*M* = 16.93 years; *SD* = 0.71 years) in enzymology. They attended two comprehensive schools (“*Gesamtschule*”) in North Rhine-Westphalia (Germany). Two biology school classes (*n* = 60 students; 75% female) were taught at an OSL at a North Rhine-Westphalian university and three biology school classes (*n* = 59 students; 66% female) were taught in their regular subject-specific classrooms at school.

### Study Design

**Figure 2:**
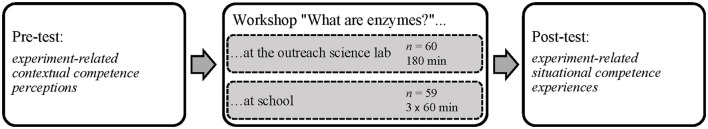
Study design.

Our study followed a quasi-experimental study design with an OSL treatment and a school treatment (see Figure 2; section Educational Program and Treatments). First, students' experiment-related contextual competence perception was assessed in pre-test. Subsequently, the students took part in a workshop addressing enzymology. In both treatment conditions, the workshop lasted 180 mins and entailed the same three experiments. In contrast with the workshop at the OSL, the workshop at school was integrated into regular biology lessons and was therefore divided into three 60-mins lessons. After the workshop, students' experiment-related situational competence experience was assessed in post-test.

### Measures

In this study, we adapted two versions of Damerau's (2012) scales about students' self-assessed experiment-related competence (see also Franken et al., 2020; Beudels et al., 2022) to assess their perceived competence in experimentation. The version used before the workshop assessed experiment-related competence perceptions on a contextual level. The version applied after the workshop evaluated the competence experiences during experimentation on a situational level. As students' experiences during a single, short-term OSL visit cannot be expected to have a substantial impact on habitual forms of their perceived competence (see Itzek-Greulich and Vollmer, 2017; Nickolaus et al., 2018), the contextual competence perception scale was not used again after the workshop. Both versions of Damerau's (2012) scales consisted of three subscales that address planning, conducting, and evaluating an experiment as depicted in the model of experimental competence from Schreiber et al. (2009, 2014); see also Franken et al. (2020). Since students did not plan the experiments in our workshop, we did not use the respective subscale in our study. The subscale *conducting an experiment* refers to the aspects of operating laboratory equipment and following experimental instructions. The items of the subscale *evaluating an experiment* refer to the aspects of evaluating data and interpreting results. The scales can be found in Table 1. All items were evaluated with a 5-point rating scale (0 = “not true at all” to 4 = “completely true”). The reliability of the scales was estimated using Cronbach's alpha, which indicated sufficient internal consistency was present (Kline, 2011).

**Table 1:** Test instruments with translated and adapted items and internal consistency (Cronbach's alpha).

**Scale**	**Cronbach's alpha**
*Pre-test: Experiment-related contextual competence perceptions*	
“Please answer these questions about experimenting.”	
-*Conducting an experiment*	α = 0.82
“I have no knack for conducting experiments.” (R)	
“I am good at using experimental equipment.”	
“I struggle with writing down experimental observations.” (R)	
“It is easy for me to set up experiments.”	
“I feel competent in conducting experiments.”	
“I think I can operate experimental equipment properly.”	
-*Evaluating an experiment*	α = 0.73
“It is easy for me to evaluate results from an experiment.”	
“I often struggle with interpreting results from experiments.” (R)	
“I think I can interpret experimental observations very well.”	
“I am good at evaluating results from an experiment.”	
*Post-test: Experiment-related situational competence experiences*	
“Please answer these questions about experimenting in the previous workshop/teaching unit about enzymes.”	
-*Conducting an experiment*	α = 0.78
“I was good at conducting the experiments.”	
“I had a knack for conducting the experiments.”	
“I had problems using the experimental equipment.” (R)	
“I struggled with writing down experimental the observations.” (R)	
“It was easy for me to set up the experiments.”	
“I think I have operated the experimental equipment very well.”	
-*Evaluating an experiment*	α = 0.72
“It was difficult for me to evaluate the results of the experiments.” (R)	
“I struggled with interpreting the results from the experiments.” (R)	
“I think I have interpreted the experimental observations very well.”	
“I was good at evaluating the results from the experiments.”	

*R, reversed items*.

### Educational Program and Treatments

In this study, all students attended the same experimental workshop under the conditions of the respective learning environment (e.g., material available at laboratories or schools). The experiments refer to the topics *enzymes as biocatalysts, temperature dependence, pH dependence, substrate specificity* and *competitive inhibition* (see Ministerium für Schule und Weiterbildung des Landes Nordrhein-Westfalen, 2014). We slightly adapted the following experiments from already available material on the educational server of the Institut für Bildungsanalysen Baden-Württemberg (n.d.). The first experiment addressed the catalysis of starch degradation by the enzyme amylase (α-amylase extracted from *Aspergillus oryzae*). Three 1% starch solutions were stained with Lugol's iodine and treated with amylase, saliva, and water (blank sample). The blue coloration of the mixtures containing amylase or saliva should have decreased; the coloration of the mixture containing water should not have changed. The decrease in the coloration indicates the degradation of starch catalyzed amylase. The second experiment dealt with the temperature and pH dependence of the enzyme catalase (extracted from *Saccharomyces cerevisiae*). Catalase was exposed for 10 mins to different temperatures (0, 20, 37, 80°C) and treated with different pH using hydrochloric acid (1%), water, and caustic soda (1%) for 5 mins. After each treatment, students added hydrogen peroxide (10%) to the enzyme suspensions. Foam columns of different heights should have been formed in the test tubes depending on the temperature and the pH. The height of the foam columns was correlated with the enzyme activity. The third experiment regarded the substrate specificity and the competitive inhibition of the enzyme urease (extracted from *Canavalia gladiata*). In the first part (substrate specificity), the students stained two urea solutions (2%) and one methyl urea solution (2%) with phenolphthalein (0.1%) and added urease. One urea solution was not treated and used as a blank sample. Different decolorization rates of the samples were observed and interpreted with regard to the lock and key principle. In the second part (competitive inhibition), the students stained a further urea solution (2%) and a solution with both urea (2%) and methyl urea (5%) using phenolphthalein (0.1%). After adding urease, different decolorization rates of the samples were observed and interpreted regarding competitive inhibition.

In both treatments, we organized the workshop as follows. The workshop was planned as an introduction to a subsequent series of lessons on enzymology; this was to ensure that all participating school classes had not been taught in enzymology in their previous 11th grade biology lessons and that the participating students possessed the same level of knowledge in this field. The experiments were organized in workstations. The students conducted the experiments in groups of three or four students. Working in groups at workstations is suitable for performing hands-on experiments (see Hofstein and Lunetta, 2004) and constitutes a common practice at OSLs (e.g., Affeldt et al., 2015; Goldschmidt and Bogner, 2016) and in schools (e.g., Schaal and Bogner, 2005; Hummel et al., 2012). Each student received a script that contained all relevant information about the structure, content, and experiments involved in the workshop. Scripts (e.g., stapled worksheets, an advanced organizer, and a laboratory guide) are suitable for documenting learning content and structuring workshops (see Hofstein and Lunetta, 2004; DeWitt and Storksdieck, 2008; Wüst-Ackermann et al., 2018). The treatments were conducted by four preservice teachers. Two preservice teachers were in the sixth semester and two preservice teachers were in the seventh semester of their study path. Three of them were randomly selected to conduct the school treatment; the preservice teacher who did not conduct the school treatment was in the sixth semester. Their acting in both treatments was not evaluated or graded. In both treatments, the preservice teachers supported the students when needed. To ensure that the students had sufficient freedom to do the experiments on their own, the following guidelines were used (see Schmidt-Weigand et al., 2008; Kersaint et al., 2011; Scharfenberg and Bogner, 2013, 2019). The students first received a prompt [e.g., “Remember, what must be considered when using (micro-)pipettes?”] when they asked for help or when interventions were necessary, such as if the laboratory equipment was not used appropriately. If a prompt was not sufficient, a further explanation or a specific instruction was provided (e.g., on the use of pipettes). The preservice teachers were trained beforehand to follow these guidelines. The regular in-service teachers did not lead the workshop to avoid possible confounding effects. Their familiarity with the students and professional knowledge could lead to a bias in the school treatment. In-service teachers often aim to experiment as successfully as possible (Abrahams and Millar, 2008; Abrahams and Reiss, 2012) and “may manipulate classroom science to obtain the expected results” (Hanauer et al., 2006, p. 1880). Moreover, in contrast to preservice teachers, they usually do not conduct the laboratory workshops at an OSL (section Non-formal Learning at Outreach Science Labs).

In the OSL treatment, the workshop was conducted by each school class as a half-day field trip to a university laboratory during which the students performed experiments for 180 mins. In the school treatment, the experimentation time was 180 mins as well. The students performed the experiments in a three-lesson teaching unit that was adapted to the schools' timetables and lesson times of each participating school class (section Study Design; Figure 2). The workshop was conducted in the regular biology classroom of the school. However, the treatments did not only differ in the location where the workshop took place. Since an OSL is not simply a classroom outside of school (section The Effects of an Outreach Science Lab on Students' Perceived Competence), we varied other characteristics; as was described in the literature (e.g., Scharfenberg, 2005; Garner et al., 2014; Sommer et al., 2018, 2020; Scharfenberg et al., 2019; Euler and Schüttler, 2020). Accordingly, the environment of an OSL is also shaped by the materials and the supervisors. Regarding materials, we used laboratory equipment and materials that are used in professional scientific laboratory work (e.g., micropipettes or water baths with a built-in thermostat and tube rack) to conduct the experiments in the OSL treatment. Research-relevant or -identical devices are part of an OSL's equipment (Scharfenberg, 2005; Schüttler et al., 2021). The students wore laboratory coats, gloves, and safety glasses since they were working in a scientific laboratory. In contrast, at school, we used equipment and materials that are usually available at this place, such as less expensive one-way pipettes instead of microliter pipettes, to conduct the experiments. Laboratory equipment is usually unavailable at schools due to its high costs (Scharfenberg and Bogner, 2013; Garner et al., 2014). The students at school were only required to wear coats, gloves, and safety glasses when working directly with hazardous materials, such as when pipetting hydrochloric acid. Regarding the supervisors, two student groups were supervised by one preservice teacher who was called a “tutor” at the OSL. At school, one whole class was supervised by one preservice teacher who was called a “teacher”. The students' performance was not graded at the OSL. At school, the regular teachers observed and graded their students.

### Data Analysis

Preliminarily, a mutlivariate analysis of variances was applied to examine whether students' situational competence experience in conducting and evaluating experiments differed between groups taught by different supervisors at each location. Additionally, we calculated Pearson correlation coefficients between the investigated variables to test whether students' situational competence experience while conducting and evaluating experiments was correlated with their respective contextual competence perception and the treatment.

To test our hypotheses, we performed two moderation analyses using the PROCESS macro (Model 1) in SPSS (see Field, 2018; Hayes, 2018). This procedure is based on a multiple linear regression model (Hayes, 2018). In both models, we estimated the effects of the independent variables *contextual competence perception* and *treatment* (0 = school; 1 = OSL), as well as their interaction (contextual competence perception × treatment) on the dependent variable *situational competence experience*. Here, the interaction coefficient quantifies how the effect of students' contextual competence perception on their situational competence experience differs between the OSL and school (see Hayes, 2018). That is, a significant interaction effect indicates whether the assumed relationship between contextual and situational perceived competence is moderated by the learning environment (see Field, 2018). In addition to *p*-values, we determined 95% confidence intervals using bootstrapping with 10,000 iterations to estimate the significance of the effects. Furthermore, conditional regression coefficients (simple slope analysis) were calculated to analyze the interaction in more detail. The conditional regression coefficients correspond to the slopes of the conditional regression lines (Field, 2018; Hayes, 2018). They depict the changes in the situational competence experience when contextual competence perception increases by the value of one standard deviation separately for the OSL treatment and the school treatment (see Hayes, 2018). The standard error estimation procedure (HC3 method) used is robust against the violation of the homoscedasticity assumption (Long and Ervin, 2000). As the moderating variable was dichotomous and the scaling of both the independent and dependent variables were the same, mean centering was omitted (Hayes, 2018). Finally, simple effects of the learning environment (*W*) on students' situational competence experience for lower and higher^1^ contextual competence perception (*X*) were analyzed “by reversing the roles of *X* and *W* in the PROCESS command” (Hayes, 2018, p. 300).

## Results

Preliminarily, no differences in students' situational competence experiences between the groups taught by different supervisors at each location were found (OSL: *F*_(2, 57)_ = 0.08, *p* = 0.927; school: *F*_(4, 112)_ = 0.31, *p* = 0.872). Correlations between the independent and dependent variables were tested and can be found in Table 2.

**Table 2:** Descriptive statistics and correlations of the investigated variables regarding conducting (below the diagonal) and evaluating the experiments (above the diagonal).

	**Conducting experiments**		**Evaluating experiments**				
**Variables**	***M***	***SD***	***M***	***SD***	**1**	**2**	**3**
1 Treatment	–	–	–	–	–	−0.15	0.20*
2 Contextual competence perception (pre-test)	2.48	0.63	2.22	0.68	−0.14	–	0.44***
3 Situational competence experience (post-test)	2.65	0.66	2.33	0.73	0.19*	0.32***	–

*N = 119; Treatment: 0 (school), 1 (outreach science lab); Contextual competence perception and situational competence experience range from 0 (strongly disagree) to 4 (strongly agree);*

* *p <0.05,*

*** *p <0.001*.

To test whether the effect of students' experiment-related contextual competence perceptions on their situational competence experiences during experimentation was moderated by the learning environment, we performed two moderation analyses: one regarding conducting the experiments and the other regarding evaluating the experiments. Both models were found to be suitable for identifying predictors of students' situational competence experience (Table 3). Specifically, the interaction effects of contextual competence perception and the treatment were found to be significant. The coefficients indicate that the effects of students' contextual competence perception on their situational competence experience were weaker at the OSL than at school.

**Table 3:** Results of the multiple linear regression analyses regarding conducting and evaluating experiments.

**Situational competence experience during**	**Estimate**	***SE***	**95% CI**		***p***
			***LL***	***UL***	
*Conducting experiments (post-test): R^2^* = 0.21, *F*_(3, 115)_ = 11.09, *p* <0.001					
Constant	0.84	0.35	0.15	1.53	0.018
Contextual competence perception (CCP; pre-test)	0.65	0.13	0.40	0.91	<0.001
Treatment	1.47	0.50	0.47	2.46	0.004
Interaction (CCP × treatment)	−0.45	0.19	−0.83	−0.08	0.018
*Evaluating experiments (post-test): R^2^* = 0.28, *F*_(3, 115)_ = 11.00, *p* <0.001					
Constant	0.41	0.38	−0.35	1.17	0.286
Contextual competence perception (CCP; pre-test)	0.80	0.16	0.49	1.11	<0.001
Treatment	1.39	0.46	0.49	2.29	0.003
Interaction (CCP × treatment)	−0.51	0.19	−0.89	−0.13	0.010

*N = 119; CI, confidence interval; LL, lower limit; UL, upper limit; Treatment: 0 (school), 1 (outreach science lab); Contextual competence perception and situational competence experience range from 0 (strongly disagree) to 4 (strongly agree)*.

To investigate the moderating effect of the treatment in more detail, simple slope analyses were performed. Regarding conducting experiments, students' contextual competence perception did not predict their situational competence experience at the OSL (Table 4). In contrast, the prediction was significant in school (Table 4). The higher students' contextual competence perception, the higher their situational competence experience. Regarding evaluating experiments, students' contextual competence perception predicted their situational competence experience in both learning environments (Table 4). The higher students' contextual competence perception, the higher their situational competence experience.

**Table 4:** Estimation of the conditional effects of students' contextual competence perception (pre-test) and treatment on the situational competence experience (post-test).

	**Cond. effect**	***SE***	**95% CI**		***p***
			***LL***	***UL***	
*Conducting experiments*					
Contextual competence perception					
At the outreach science lab	0.20	0.14	−0.08	0.47	0.155
At school	0.65	0.13	0.40	0.91	<0.001
Treatment					
Lower contextual competence perception (Mean – *SD*)	0.62	0.18	0.28	0.97	<0.001
Higher contextual competence perception (Mean + *SD*)	0.06	0.15	−0.23	0.34	0.708
*Evaluating experiments*					
Contextual competence perception					
At the outreach science lab	0.29	0.12	0.06	0.52	0.014
At school	0.80	0.16	0.49	1.11	<0.001
Treatment					
Lower contextual competence perception (Mean – *SD*)	0.60	0.19	0.24	0.97	<0.001
Higher contextual competence perception (Mean + *SD*)	−0.09	0.17	−0.43	0.25	0.612

*N = 119; Contextual competence perception range from 0 (strongly disagree) to 4 (strongly agree) was assessed in pre-test (conducting experiments: M = 2.48, SD = 0.63; evaluating experiments: M = 2.22, SD = 0.68)*.

**Figure 3:**
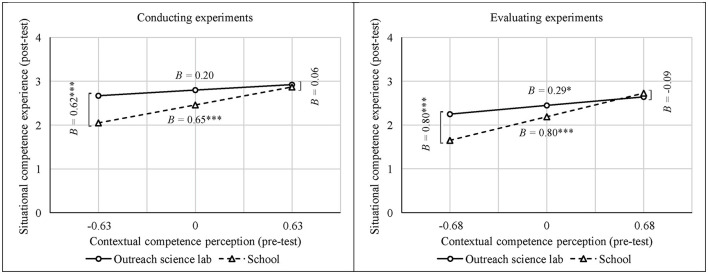
Students' situational competence experiences (post-test) while conducting (left) and evaluating experiments (right) depending on their contextual competence perceptions (pre-test) in conducting and evaluating experiments in the treatments outreach science lab and school (^*^p < 0.05, ^***^p < 0.001).

Figure 3 visualizes the interaction effects. The slope of the regression lines for the school treatment were steeper than the slopes of the regression lines for the OSL treatment. This pattern illustrates a stronger correlation between students' contextual perceptions and situational experiences of competence at school than at the OSL (see also Table 4). Moreover, Figure 3 illustrates that students' situational competence experiences differed between the treatments when their respective contextual competence perception was lower (Mean – *SD*). Indeed, these students felt more competent during experimentation at the OSL than at school (Table 4). On the other hand, when contextual competence perceptions were higher (Mean + *SD*), the students felt competent during experimentation to the same extent at both places of learning (Table 4).

In summary, these findings indicated that students' situational competence experience was more strongly predicted by their contextual competence perception at school than at the OSL. Additionally, the students with a lower contextual competence perception had a higher level of situational competence experience at the OSL than at school.

## Discussion

In the current study, we hypothesized that the learning environment moderates the effect of students' experiment-related contextual competence perceptions on their situational competence experiences during experimentation. The findings of the analyses were consistent with our expectations. We found significant interactions between the investigated predictors. These findings support the moderating role of the learning environment in the relationship between contextual perception and situational experience of competence during conducting (H_1_) and evaluating experiments (H_2_). For both phases of experimentation, the effect of students' contextual competence perception on their situational competence experience was stronger at school than at the OSL. In the school treatment, there was a significant effect for both conducting and evaluating experiments. In the OSL treatment, we found different relationships. When conducting experiments, there was no effect. That is, students with a higher contextual competence perception did not experience themselves as more competent when conducting the experiments than students with a lower contextual competence perception. However, regarding the evaluation of experiments, the students' contextual competence perception predicted their situational competence experience. Compared to the rather cognitive activities in the evaluation of experiments (see Schreiber et al., 2009), the practical activities when students conducted the experiments may have provided intense experiences of competence (see Hofstein and Lunetta, 2004; Tal and Dallashe, 2019; Tsybulsky, 2019), particularly at the OSL (see Itzek-Greulich and Vollmer, 2017). This circumstance might have contributed to a lower correlation between students' contextual and situational perceived competence in conducting experiments over evaluating experiments. Accordingly, Damerau (2012) found a stronger correlation between students' self-assessed and experienced experiment-related competencies in evaluating experiments than in conducting experiments.

In addition, comparing students' situational competence experiences between the two learning environments, the investigated effects are in favor of the students who had a lower contextual competence perception and then visited the OSL. These students experienced themselves as more competent than students with a lower contextual competence perception at school. Those with a higher contextual competence perception had a comparable situational experience of competence in both learning environments. Compared to students who experimented at school or had a higher contextual competence perception, this finding reveals that students who perceived themselves as generally less competent benefited most in terms of their situational competence experience through the performance of experiments at the OSL. Previous studies have shown similar findings on student motivation. For instance, they have revealed that less interested students (e.g., Pawek, 2009; Glowinski and Bayrhuber, 2011; Damerau, 2012) and low achieving students (Itzek-Greulich and Vollmer, 2017) benefited most from attending an OSL in terms of effects on their motivational outcomes. In general, low achievers exhibit low interest (Hong and Lin, 2011), which is strongly correlated with students' self-concept of abilities, especially in higher grades (Marsh et al., 2005; Möller and Trautwein, 2015). They rate their skills low and feel less competent at a contextual level or within a specific domain (Hughes et al., 2011). For these students, support can be particularly helpful (see Vygotsky, 1978; Schmidt-Weigand et al., 2008; Itzek-Greulich and Vollmer, 2017, 2018; Großmann and Wilde, 2019). In the current study, the support was provided by the tutors and teachers (section Educational Program and Treatments). Their support might have helped the students with a lower contextual competence perception to feel more confident in experimentation and to perceive themselves as effective or successful throughout that process (see Schmidt-Weigand et al., 2008; Arnold et al., 2017; Großmann and Wilde, 2019). The students received support to operate the laboratory equipment successfully during the OSL visit. The laboratory equipment (e.g., microliter pipettes) is of higher quality than the equipment that is usually available at schools (e.g., one-way pipettes). It is possible that successfully operating the better laboratory materials might have led to a higher experience of effectiveness in the OSL than successfully operating the materials at school (see Pawek, 2009; Schüttler et al., 2021). In addition, the support at the OSL was provided more frequently than at school. The students at the OSL might have received more individual attention than the students at school, especially in situations where several groups might have needed support from their supervisor at the same time. These circumstances might explain why students with a lower contextual competence experienced themselves as more competent at the OSL than the students at school with only one teacher instructing the whole class (see Jang et al., 2010; Reeve, 2015). On the other hand, the situational competence experience of the students with a higher contextual competence perception did not differ between the treatments although there was more support available at the OSL. It may be that these students might have required less support than students who felt insecure and less competent in experimentation when working on the same task (see Vygotsky, 1978; Schmidt-Weigand et al., 2008; Großmann and Wilde, 2019). This circumstance might also explain why there were lower correlations between contextual perceptions and situational experiences of competence at the OSL compared to those in school.

Moreover, the findings could be explained by differences in the formality of learning within the two learning environments. The non-formal environment of the OSL might have contributed positively to students' social interactions (see Hofstein and Lunetta, 2004; Schwan et al., 2014; Tal and Dallashe, 2019). In addition to the tutors' support, peer assistance among the students might have helped those with a lower contextual competence perception to master the experimental tasks (see Donato, 1994; Levine and Thompson, 2004; Reicher et al., 2006). In turn, these students might have experienced themselves as more competent at the OSL than at school (see Wentzel, 2017). Furthermore, the OSL was visited as a supplement to the regular biology class, and the students' performance was not graded (section Educational Program and Treatments). Therefore, the activities at the OSL might have been less associated with grading performance than the activities at school (see Pawek, 2009) and might have been perceived “as a fresh start” (Itzek-Greulich and Vollmer, 2017, p. 18). Since OSLs emphasize the acquisition of new competencies in an assessment-free space with special respect paid to students' individual needs (see Pawek, 2009; Euler and Schüttler, 2020), the less performance-oriented atmosphere of this environment might have positively affected students' motivational experiences as well as their perceived autonomy and competence (see Meece et al., 2006; Itzek-Greulich and Vollmer, 2017; Alp et al., 2018). These results from previous studies support our findings on students' situational competence experience. Additionally, as the students' performance was not graded, asking for or receiving support would not be associated with a bad grade, but rather with informative feedback and constructive support (see Pawek, 2009). To prevent the students from feeling too closely observed, the tutors were instructed to not express controlling behaviors (see Reeve, 2015) and to ensure that the students conducted and evaluated the experiments predominantly on their own (section Educational Program and Treatments). We also explained to the students why support can sometimes be helpful and that it could be requested (e.g., in using the micropipettes). Therefore, they may have experienced being controlled less but rather supported at the OSL. If the students perceived themselves as being controlled by their tutors, their situational competence experience might have been impaired (Jang et al., 2010; Su and Reeve, 2011; Reeve, 2015; Großmann et al., 2020). However, as our analyses revealed, the students at the OSL did not have had a lower competence experience than the students at school. Therefore, the possible negative effect of the tutoring did not lead to a lower situational competence experience at the OSL compared to the school treatment.

As the regular teachers were present during the intervention, they might have had an influence as well. In both learning environments, the regular teachers observed but did not intervene in the conduction of the workshop. According to the concepts of non-formal and formal learning, the students in the OSL treatment were not graded, but the students in the school treatment were graded by their regular teachers (section Educational Program and Treatments). Grades can exert pressure on students that may have a negative effect on their perceived competence (see Su and Reeve, 2011; Reeve, 2015). The negative effect may be stronger when students' contextual competence perception is low as these students may think they are more likely to receive a lower grade (see Skaalvik and Skaalvik, 2005). It is possible that grading in the school treatment might have contributed to our results.

With regard to the interpretation and explanation of the effects found, it is essential to consider a few aspects. First, the investigated learning environments had more differences in characteristics apart from their location outside of or inside schools, such as the provision of support and the formality of learning. Therefore, the effects found cannot be attributed to a single characteristic, but rather to the sum of the characteristics that shape experimentation at an OSL or in school. Future studies could investigate such variables in more detail, for example by varying the supervisor-student-ratio at an OSL. However, this study was conducted in two authentic settings that could yield ecological validity (Lewkowicz, 2010) and provide evidence regarding the value of practical laboratory work at OSL in terms of students' situational competence experience.

Second, the sample size was not very large. Nevertheless, it was sufficiently large for the performed statistical analyses (Field, 2018). In addition, the students only came from one type of secondary schools in North Rhine-Westphalia. School type might have an impact on the effect of the learning environment (see Basten et al., 2014) and education may differ between the federal states, for instance, regarding the curriculum (Standing Conference of the Ministers of Education Cultural Affairs of the Länder in the Federal Republic of Germany, 2019). Consequently, the applicability of our results to other school types and other federal states may be limited. In future studies, a larger sample should be examined and additional types of secondary schools such as a ‘*Gymnasium*' (i.e., the highest track) in other federal states should be included.

Third, other than conducting and evaluating experiments, the planning of an experimental setup is also a phase of experimentation (Klahr, 2000; Schreiber et al., 2009). As the investigated OSL focused on conducting and evaluating experiments and the students did not plan their own experiments in the workshop, we did not investigate students' perceived competence in planning experiments. It may be interesting to investigate whether similar results can be found for planning experiments, in particular, with regard to students with a higher contextual competence perception in planning. As students generally rate their skills in planning the lowest compared to other phases of experimentation (Damerau, 2012; see also Franken et al., 2020), the provision of support during this phase of experimentation may also be important for students with a higher contextual competence perception in terms of their situational competence experience. Moreover, further variables, such as prior knowledge, may have an influence on students' perceived competence (see Jang et al., 2010; Reeve, 2015; Ryan and Deci, 2017). However, in a previous study (Großmann et al., 2020), students' prior knowledge could not have been confirmed as a predictor of their perceived competence during biology class. In addition, as the current study took place before the regular teachers had started to teach their students in enzymology, all participants might have shared similar prior knowledge (section Educational Program and Treatments). Nevertheless, we cannot rule out that the students might have acquired knowledge on enzymology in their previous school career.

Fourth, the novelty of an out-of-school learning environment may have negative effects on student learning (see Eshach, 2007). In our study, the students visited an OSL that was unfamiliar to them and might have perceived the environment as complex at first (see Pawek, 2009; Euler and Schüttler, 2020). Here, the students performed comprehensive experimental tasks using laboratory equipment that they were not familiar with from school (see Scharfenberg and Bogner, 2013; Garner et al., 2014; Affeldt et al., 2015). As a result, the students at the OSL might have considered the experiments initially more challenging, difficult, and extensive than the students at school did (see Pawek, 2009; Euler and Schüttler, 2020) and might have been overwhelmed when executing the experimental tasks (Damerau, 2012; Scharfenberg and Bogner, 2013). In this situation, the students' situational competence experience could have been impaired (see Jang et al., 2010; Reeve, 2015). As discussed, the provided support at the OSL might have prevented the students from being overwhelmed by an unfamiliar and complex environment (see Kirschner et al., 2006; Kersaint et al., 2011; Scharfenberg and Bogner, 2013) and might therefore have reduced a possible effect of novelty on students' situational competence experience (see Vygotsky, 1978; Mohr-Schroeder et al., 2014). In addition, our results reveal that the students at the OSL did not perceive themselves as less competent than the students who attended the workshop in their familiar science classroom at school. This result indicates that the novelty of the new environment did not lead to a lower situational competence experience at the OSL compared to the school.

In addition to novelty effects, some further aspects should be taken into consideration when visiting OSLs. As was the case in this study, most of the investigated effects for OSLs are short-term effects (Guderian and Priemer, 2008; Schütte and Köller, 2015; Nickolaus et al., 2018). In the long term, students may benefit from their experiences at an OSL in terms of habitual forms of motivation, such as individual interest or contextual competence perception, provided that the positive learning experiences of the visit could be maintained through post-visit instruction (see Itzek-Greulich et al., 2015; Itzek-Greulich and Vollmer, 2017). In addition to post-visit instruction, teachers should prepare visits to OSLs, as the effectiveness of the visit depends on the (organizational) pre-visit instruction (Orion, 1993; Wilde and Bätz, 2006; Glowinski and Bayrhuber, 2011; Itzek-Greulich and Vollmer, 2017). Therefore, an OSL visit should not be considered a separate, single event or a substitute for regular science classes (Glowinski and Bayrhuber, 2011; Itzek-Greulich et al., 2015; Euler and Schüttler, 2020). Both science classes in school and science educational outreach programs are mutually complementary ways for students to learn science. That is, linking the lessons at school with OSL visits may be desirable (Orion, 1993; Eshach, 2007; Itzek-Greulich, 2020).

In conclusion, our findings contribute to the current state of research in the field of non-formal learning's impact on student motivation by investigating the interrelationship of students' competence perceptions and experiences during experimentation at an OSL and at school. They highlight great potential for fostering students' situational motivation in the context of out-of-school learning. In particular, the students who previously felt less competent in experimentation could benefit from performing experiments at an OSL in terms of their situational competence experience. Performing experiments at an OSL seems to provide positive situational experiences of one's own competence, almost independently of a more general perception of competence. This finding is in line with the assumption that an OSL visit contributes to the development of positive qualities of student motivation (Garner et al., 2014; Affeldt et al., 2015; Scharfenberg et al., 2019; Euler and Schüttler, 2020); it also aligns with findings from previous studies that less motivated students may especially benefit from attending a workshop at an OSL (e.g., Glowinski and Bayrhuber, 2011; Damerau, 2012; Itzek-Greulich and Vollmer, 2017). Our findings suggest that OSLs offer suitable conditions for addressing student heterogeneity in terms of their perceived competence.

## Data Availability Statement

The raw data supporting the conclusion of this article will be made available by the authors, without undue reservation. Requests to access the datasets should be directed to TK, tim.kirchhoff@uni-bielefeld.de.

## Ethics Statement

The studies involving human participants were reviewed and approved by Ethik-Kommission der Universität Bielefeld. Written informed consent from the participants' legal guardian/next of kin was not required to participate in this study in accordance with the national legislation and the institutional requirements.

## Author Contributions

TK developed the concept of and design for the study, recruited the sample, performed the statistical analysis, and wrote the first draft of all article sections. MW contributed to the study design and reviewed the article. NG contributed to the study design and the statistical analyses and reviewed the article. All authors agree to be accountable for the content of the work. All authors have contributed, read, and approved the submitted manuscript.

## Funding

This project is part of the Qualitätsoffensive Lehrerbildung, a joint initiative of the Federal Government and the *Länder* which aims to improve the quality of teacher training. The programme is funded by the Federal Ministry of Education and Research (Funding Code: 01JA1908). We acknowledge support for the publication costs by the Open Access Publication Fund of Bielefeld University and the Deutsche Forschungsgemeinschaft (DFG). The authors are responsible for the content of this publication.

## Conflict of Interest

The authors declare that the research was conducted in the absence of any commercial or financial relationships that could be construed as a potential conflict of interest.

## Publisher's Note

All claims expressed in this article are solely those of the authors and do not necessarily represent those of their affiliated organizations, or those of the publisher, the editors and the reviewers. Any product that may be evaluated in this article, or claim that may be made by its manufacturer, is not guaranteed or endorsed by the publisher.

## Footnotes

^1^The terms “lower” and “higher” contextual competence perception refer to levels of contextual competence perception of at least one standard deviation (conducting experiments: *SD* = 0.63; evaluating experiments: *SD* = 0.68) below or above the mean of students' contextual competence perception (conducting experiments: *M* = 2.48; evaluating experiments: *M* = 2.22).
